# A New Formula Consisting of the Initial Independent Predictors of All-Cause Mortality Derived from a Single-Centre Cohort of Antineutrophil Cytoplasmic Antibody-Associated Vasculitis

**DOI:** 10.3390/jcm14030779

**Published:** 2025-01-25

**Authors:** Pil Gyu Park, Jiyeol Yoon, Yong-Beom Park, Sang-Won Lee

**Affiliations:** 1 Division of Rheumatology, Department of Internal Medicine, National Health Insurance Service Ilsan Hospital, Goyang 10444, Republic of Korea; pilgyupark@nhimc.or.kr; 2 Division of Rheumatology, Department of Internal Medicine, Yonsei University College of Medicine, Seoul 03722, Republic of Korea; 3 Institute for Immunology and Immunological Diseases, Yonsei University College of Medicine, Seoul 03722, Republic of Korea

**Keywords:** antineutrophil cytoplasmic antibody-vasculitis, mortality, formula, five-factor score, predict

## Abstract

**Objective:** In this study, we develop a new formula for predicting all-cause mortality in an ethnicity/region-specific cohort of antineutrophil cytoplasmic antibody-associated vasculitis (AAV). **Methods:** We included 290 Korean patients with AAV in this study and retrospectively reviewed their medical records regarding clinical data at diagnosis and during follow-up. We introduce a new index, called the NFPM value after the initials of New Formula for Predicting Mortality, which we derived using the independent predictors of all-cause mortality obtained in the multivariable Cox proportional hazard analysis. The cut-offs of the parameters for mortality were determined using the highest or lowest tertile of each parameter according to its positive or negative association with all-cause mortality, respectively. **Results:** The median age was 60.0 years and 35.9% were male patients. Of the 290 patients, 39 died during follow-up (13.4%). In the multivariable Cox analysis, male sex, the five-factor score (FFS), and serum albumin were independent predictors of all-cause mortality. A new formula was developed as follows: NFPM = male sex (yes = 1 or no = 0) + FFS ≥ 2.0 (yes = 1 or no = 0) + serum albumin ≤ 3.2 mg/dL (yes = 1 or no = 0). We demonstrated that patients with a NFPM value ≥ 2 seemed to have an increased risk for all-cause mortality compared to those with a NFPM value < 2. **Conclusions:** This study demonstrated that it could be clinically useful and significant to develop a new formula to predict all-cause mortality using independent predictors in each different ethnicity/region-specific cohort of AAV.

## 1. Introduction

Antineutrophil cytoplasmic antibody (ANCA)-associated vasculitis (AAV) is characterised by necrotising vasculitis influencing primarily small-sized vessels and occasionally medium-sized arteries. AAV has three subtypes, microscopic polyangiitis (MPA), granulomatosis with polyangiitis (GPA), and eosinophilic GPA (EGPA). MPA often affects the kidneys and lungs, and GPA mainly provokes inflammation in the upper and lower respiratory tracts. Meanwhile, EGPA primarily exhibits clinical manifestations based on allergic backgrounds [1,2,3]. In patients with severe or life-threatening cases, such as diffuse alveolar haemorrhage, progressive rapid glomerulonephritis, central/peripheral nervous system vasculitis, and cardiac involvement, the mortality rate may increase at a higher rate than those in other systemic vasculitides, and thus remission induction therapies are strongly recommended [4,5]. However, despite treatment, the mortality rate in patients with AAV has been known to be 24.3% and 26.9% [6,7]. Additionally, in a cohort of Korean patients with AAV, 13.2% of the patients died during follow-up [8].

Some studies have also investigated initial biomarkers or indices for predicting all-cause mortality during the follow-up period [9,10,11], in addition to traditional risk factors for mortality [12]. However, because some of these parameters need to be measured again, they may only be suitable for prospective—but not retrospective—studies [11]. The advantage of retrospective studies is that based on their results, the risk for all-cause mortality in patients newly diagnosed with AAV may be assessed, which also provides an opportunity to better mitigate/manage poor outcomes in the future. Therefore, to address this problem, the present study aimed to develop a new formula for predicting all-cause mortality in an ethnicity/region-specific cohort of AAV using the results of tests routinely performed in clinical practice.

## 2. Materials and Methods

### 2.1. Patients

In this study, according to the inclusion criteria, we selected 160, 74, and 56 patients with MPA, GPA, and EGPA from the Severance Hospital ANCA-associated vasculitides (SHAVE) cohort, a cohort of Korean patients with AAV, and we reviewed their medical records. The SHAVE cohort was established in 2016 and retrospectively included patients who had been diagnosed with AAV from 2000 until then, and prospectively, those with AAV who were diagnosed with AAV from 2016 until 2023. The inclusion criteria were (i) patients that were initially diagnosed with MPA, GPA, and EGPA in this tertiary university hospital from 2000 to 2023; (ii) they met the 1990 American College of Rheumatology (ACR) classification criteria for AAV [13,14], the 2007 European Medicine Agency (EMA) algorithm for AAV [1], and the revised 2012 Chapel Hill Consensus Conference nomenclature of systemic vasculitides [2]; (iii) they were further reclassified as having AAV according to the recently proposed 2022 ACR/European Alliance of Associations for Rheumatology (EULAR) classification criteria for AAV [3,15,16,17]; (iv) they had medical records that were sufficiently well written for collecting clinical, laboratory, radiological, and histological data at AAV diagnosis and during follow-up; (v) they had been followed up for 3 months or longer after AAV diagnosis; (vi) they had no serious medical conditions mimicking AAV such as malignancies and severe infectious diseases requiring hospitalisation at AAV diagnosis [15,16,17]; (vii) they had no medical or drug history affecting ANCA positivity before AAV diagnosis; and (viii) they were not exposed to immunosuppressive drugs for AAV treatment within 4 weeks before AAV diagnosis. The present study was approved by the Institutional Review Board (IRB) of Severance Hospital (Seoul, Korea, IRB No. 4-2020-1071) and was conducted according to the Declaration of Helsinki. Given the retrospective design of the study and the use of anonymised patient data, the requirement for written informed consent was waived.

### 2.2. Clinical and Laboratory Data

In terms of data at AAV diagnosis, demographic data included age, sex, and body mass index. AAV subtype, ANCA type, and AAV-specific indices, the Birmingham Vasculitis Activity Score (BVAS), and the Five-Factor Score (FFS) were collected as AAV-related data. The BVAS has nine sub-items according to the affected systemic manifestations and the total score ranges from 0 to 63 [18]. Alternatively, the FFS is based on the five parameters including age above 65 years; cardiac involvement; gastrointestinal involvement; renal insufficiency; and absence of ear, nose, and throat symptoms [19]. Myeloperoxidase (MPO)-ANCA and proteinase 3 (PR3)-ANCA were measured by an immunoassay, and perinuclear (P)-ANCA and cytoplasmic (C)-ANCA were detected by an indirect immunofluorescence assay. Both the results for MPO-ANCA or PR3-ANCA and P-ANCA or C-ANCA were recognised as ANCA results based on the 2022 ACR/EULAR classification criteria for AAV [15,16,17]. Type 2 diabetes mellitus, hypertension, and dyslipidaemia—traditional risk factors for mortality in the general population—were also recorded as comorbidities [12]. The initial laboratory data included the results of tests that were routinely performed in real clinical practice such as the erythrocyte sedimentation rate (ESR), C-reactive protein (CRP), white blood cell and platelet counts, haemoglobin, blood urea nitrogen, serum creatinine, total protein, and albumin levels. In terms of data during follow-up, the number of deceased patients, the follow-up duration based on all-cause mortality, and the medications administered were investigated. All-cause mortality was defined as the death of any cause, and the follow-up durations based on all-cause mortality were defined as the period from AAV diagnosis to death among deceased patients or the period from AAV diagnosis to the last visit among surviving patients.

### 2.3. Developing a New Formula for Predicting All-Cause Mortality

We named the new index, NFPM, after the initials of New Formula for Predicting Mortality and developed it in the following three orders: first, the independent predictors of all-cause mortality were obtained using the multivariable Cox proportional hazard analysis; second, regarding a categorical independent predictor, one point was assigned to a case with the presence of the variable; and third, regarding a continuous independent predictor, the lower limit of the highest tertile of the variable with the positive association with all-cause mortality, or the higher limit of the lowest tertile of the variable with the negative association with all-cause mortality was first determined, and one point was assigned to a case belonging to the highest tertile of the variable.

### 2.4. Statistical Analyses

All statistical analyses were performed using IBM SPSS Statistics for Windows, version 26. Categorical and continuous variables were expressed as numbers (percentages), and median (25 and 75 percentiles), respectively. The multivariable Cox proportional hazard analysis using variables with statistical significance in the univariable analysis was used to determine the predictors of all-cause mortality during follow-up. The comparison of the cumulative survival rates between the two groups was carried out using the Kaplan–Meier survival analysis with the log-rank test. The relative risk (RR) of the cut-off for all-cause mortality was analysed using contingency tables and the chi-square test. Statistical significance was considered at *p* < 0.05.

## 3. Results

### 3.1. Characteristics of 290 Patients with AAV

Regarding variables at diagnosis, the median age was 60.0 years, and 104 (35.9%) were male patients. Of the 290 patients, 160, 74, and 56 were classified as having MPA, GPA, and EGPA, respectively. MPO-ANCA (or P-ANCA) and PR3-ANCA (or C-ANCA) were positive in 198 (68.3%) and 48 (16.6%) patients, respectively. ANCA was detected in 206 patients (88.0%). The median BVAS, FFS, ESR, and CRP were 12.0, 1.0, 59.5 mm/h, and 13.8 mg/L, respectively. A total of 77, 116, and 59 patients had type 2 diabetes mellitus, hypertension, and dyslipidaemia, respectively. The remaining laboratory results are described in Table 1. Regarding variables during follow-up, 39 of the 290 patients (13.4%) died during the median follow-up duration of 47.2 months. Glucocorticoids were given to 282 patients (97.2%). Among immunosuppressive drugs, cyclophosphamide (55.5%) was the most frequently administered, followed by azathioprine (53.1%) (Table 1).

### 3.2. Initial Independent Predictors of All-Cause Mortality

In the univariable Cox analysis using variables at AAV diagnosis, age (HR 1.062, *p* < 0.001), male sex (HR 2.633, *p* = 0.003), body mass index (HR 1.121, *p* = 0.011), BVAS (HR 1.082, *p* < 0.001), FFS (HR 1.960, *p* <0.001), ESR (HR 1.009, *p* = 0.036), CRP (HR 1.008, *p* = 0.001), white blood cell count (HR 1.000, *p* = 0.036), haemoglobin (HR 0.790, *p* = 0.002), blood urea nitrogen (HR 1.011, *p* = 0.004), serum creatinine (HR 1.143, *p* = 0.026), serum total protein (HR 0.562, *p* = 0.003), and serum albumin (HR 0.364, *p* < 0.001) were significantly associated with all-cause mortality. In the multivariable Cox analysis, male sex (HR 3.457, 95% confidence interval [CI] 1.644, 7.270), FFS (HR 1.655, 95% CI 1.094, 2.504), and serum albumin (HR 0.354 95% CI 0.174, 0.720) were independently associated with all-cause mortality. Alternatively, BVAS (HR 1.028, 95% CI 0.969, 1.091) was not independently associated with all-cause mortality. Therefore, male sex, the FFS, and serum albumin were determined as the initial independent predictors of all-cause mortality in patients with AAV and were included in the NFPM as the parameters (Table 2).

### 3.3. Developing a NFPM Among the 290 Patients with AAV

As mentioned in Section 2, for all-cause mortality, the lower limit of the highest tertiles of the FFS, and the higher limit of the lowest tertile of serum albumin were determined as 2.0 and 3.2 mg/dL, respectively. A categorical variable of FFS ≥2.0 is currently used in predicting poor prognoses of AAV. Whereas, to develop NFPM in this study, a continuous variable of the sum of FFS was selected, and coincidentally, the lower limit of FFS was identified as the same as FFS ≥ 2.0. Therefore, the NFPM among the 290 patients with AAV was developed as follows:

**NFPM (Korean patients with AAV) = male sex (yes = 1 or no = 0) + FFS ≥ 2.0 (yes = 1 or no = 0) + serum albumin ≤ 3.2 mg/dL (yes = 1 or no = 0).**

NFPM values range from 0 to 3.

### 3.4. Comparison of Cumulative Patients’ Survival Rates According to the Values of NFPM

**Figure 1:**
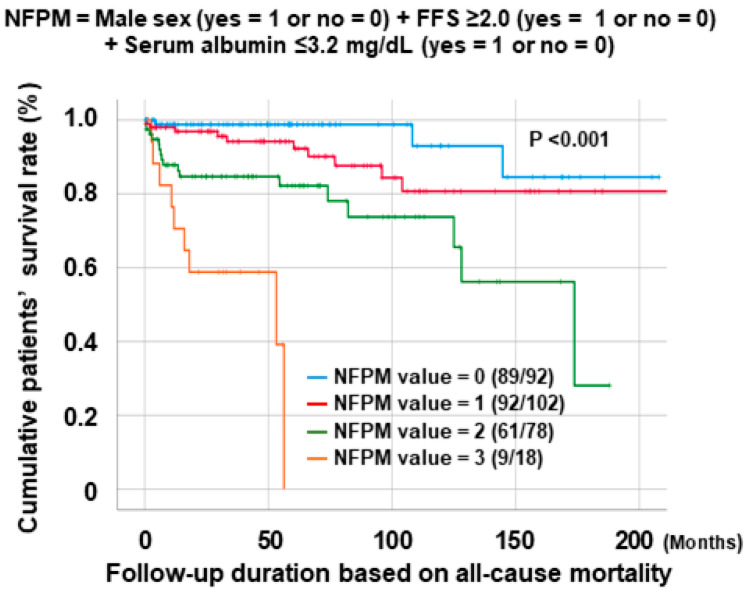
Comparison of cumulative patients’ survival rates among the four NFPM values. NFPM: new formula for predicting all-cause mortality; FFS: five-factor sore.

Among the 290 patients with AAV, 92 (31.7%), 102 (35.2%), 78 (26.9%), and 18 (6.2%) patients had NFPM values of 0, 1, 2, and 3, respectively. Additionally, during the follow-up periods of AAV, the cumulative patients’ survival rates exhibited significantly inverse patterns in proportion to NFPM values (96.7%, 90.2%, 78.2%, and 50.0%, *p* < 0.001) (Figure 1).

### 3.5. Determining a Cut-Off of the NFPM Value for All-Cause Mortality

**Figure 2:**
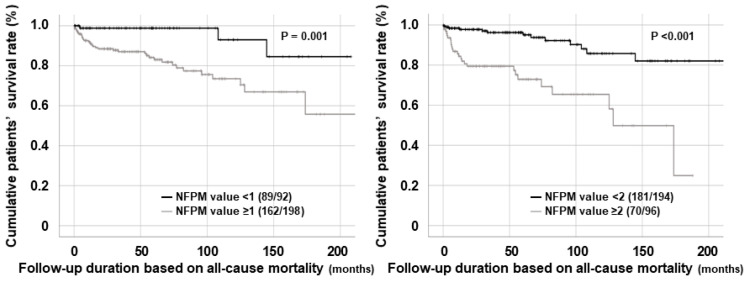
Comparison of cumulative patients’ survival rates among the two cut-offs of NFPM values. NFPM: new formula for predicting all-cause mortality.

First, when patients were divided into two groups according to a NFPM value of 1, 198 of the 290 patients were assigned to a group with a NFPM value ≥ 1. Patients with a NFPM value ≥ 1 exhibited a significantly lower cumulative patients’ survival rate than those with a NFPM value < 1 (81.8% vs. 96.7%, *p* = 0.001). Moreover, patients with a NFPM value ≥ 1 had a higher risk for all-cause mortality than those with a NFPM < 1 (RR 6.593, CI 1.974, 22.017, *p* < 0.001). Additionally, when patients were divided into two groups according to a NFPM value of 2, 96 of the 290 patients were allocated to a group with a NFPM value ≥ 2. Patients with a NFPM value ≥ 2 showed a significantly reduced cumulative patients’ survival rate compared to those with a NFPM value < 2 (72.9% vs. 93.3%, *p* < 0.001). Furthermore, patients with a NFPM value ≥ 2 seemed to have an increased risk of all-cause mortality compared to those with a NFPM value < 2 (RR 5.171, 95% CI 2.516, 10.631, *p* < 0.001) (Figure 2). Of the two cut-offs, we set the cut-off of the NFPM values for all-cause mortality as 2 for the following two reasons: The first reason was that a NFPM value of 2 was the lower limit of the highest tertile of NFPM values, which was consistent with the methodological approach of this study to obtain the cut-offs of the independent predictors. The other reason was that the lower limit of 95% CI in cases with a NFPM value ≥ 2 was 2.516, which is higher than that in cases with a NFPM value ≥ 1, despite a higher RR in cases with a NFPM value ≥ 1.

## 4. Discussion

The ultimate purpose of this study was not to propose an absolute and uniform formula consisting of a few fixed variables and correlation coefficients for predicting all-cause mortality in patients with AAV after diagnosis. Rather, it was to show how to develop and suggest a changeable and elastic formula that is composed of multiple variables and can predict all-cause mortality in patients with AAV after diagnosis. This is because in real clinical practice, a formula specific to a unique cohort with diverse ethnicities and regional characteristics is more effective. In this context, we selected 290 AAV patients from the well-controlled cohort of Korean patients with AAV and included them in the study, in which we obtained several findings as follows: First, of the 290 patients, 39 died during follow-up, which might imply the mortality rate of this cohort of Korean patients with AAV was 13.4% because it included a considerable number of patients despite being a single-centre study. Second, by the multivariable Cox proportional hazard analyses, this study demonstrated that male sex, the FFS, and serum albumin at AAV diagnosis were independent predictors of all-cause mortality during follow-up. Third, using three independent predictors of all-cause mortality, this study derived a new equation named NFPM, expressed as follows: NFPM (Korean patients with AAV) = male sex (yes = 1 or no = 0) + FFS ≥ 2.0 (yes = 1 or no = 0) + serum albumin ≤ 3.2 mg/dL (yes = 1 or no = 0). Fourth, this study revealed a significantly inverse pattern between the cumulative patients’ survival rates and NFPM values. Lastly, this study also revealed that patients with a NFPM value ≥ 2 (the highest tertile of NFPM values) seemed to have an increased risk for all-cause mortality compared to those with a NFPM value < 2. We demonstrated that it could be clinically meaningful to develop a new formula to predict all-cause mortality using independent predictors for patients with AAV from a certain race or region, where unique and specific genetic and environmental factors may differ. Therefore, we hope that the methodological approach of this study can be applied to patients with AAV from other races and regions.

The NFPM derived in this study includes three independent predictors of all-cause mortality as parameters for the newly developed formula. Each independent predictor can predict mortality. Nevertheless, a natural question may arise as to why it is necessary to derive a formula that includes three independent predictors of all-cause mortality in patients with AAV. To answer this question, we attempted two comparisons. First, one comparison was carried out to compare the *p*-values in the cumulative patients’ survival rates between the three predictors and the NFPM. The cut-offs of the four variables share common characteristic, which is that they were determined based on the highest tertile of each variable. In the comparative analysis of the cumulative patients’ survival rates, a *p*-value of NFPM value ≥ 2 (*p* = 1.3 × 10^−^^7^) was much lower than *p*-values of male sex (*p* = 1.7 × 10^−^^3^), FFS ≥ 2 (*p* = 4.6 × 10^−^^5^), and serum albumin ≤ 3.2 mg/dL (*p* = 2.5 × 10^−^^4^) (Supplementary Figure S1). Next, the other comparison was to compare the relative risks for all-cause mortality among the four variables. Similarly, a RR for all-cause mortality of NFPM value ≥ 2 (RR 5.171, 95% CI 2.516, 10.631 *p* < 0.001) was remarkably higher than RRs of male sex (RR 2.361, 95% CI 1.194, 4.672, *p* = 0.012), FFS ≥ 2 (RR 4.096, 95% CI 1.979, 8.477, *p* < 0.001), and serum albumin ≤ 3.2 mg/dL (RR 3.792, 95% CI 1.891, 7.602, *p* < 0.001). Therefore, although it may be cumbersome to derive a new formula using independent predictors of all-cause mortality, we believe that it has the advantage of being more effective and stable than a single variable in predicting all-cause mortality in patients with AAV.

From another perspective, NFPM has two additional advantages as follows: stability and diversity. First, since NFPM is a formula consisting of three multiple parameters, it is less variable than the fluctuation range of one variable. In other words, even if one of the three variables experiences a large fluctuation due to a sudden situational influence, the fluctuation range can be significantly reduced through the buffering effect of the remaining two variables, thus offering greater stability. Next, perhaps coincidentally, NFPM contains variables corresponding to all three areas of mortality risk in patients with AAV: (i) male sex as a traditional risk [12]; (ii) the FFS as an AAV-related risk [20]; and (iii) serum albumin as an inflammation-related risk [21]. In other words, it can be said to be an advantage of diversity in that it is an indicator that reflects various risk situations. In addition, there was concern about whether it might be unreasonable to use the tertile stratification of each variable as a cut-off. However, since previous clinical studies using the tertile stratification are not rare [22,23], it may be sufficiently valid, and it may have an advantage in that it is more convenient than deriving a cut-off with the maximum sum of sensitivity and specificity using the ROC curve.

Meanwhile, we were concerned that it might be impossible to know the survival or death or the follow-up duration in patients who did not visit the clinic for a significant amount of time. Thus, we reviewed the medical records, accordingly. According to a previous study [24], we defined new concepts regarding ‘patients lost-to-follow-up’ and ‘adjusted follow-up duration’ in this study. First, ‘patients lost-to-follow-up’ were defined as those who had not visited the clinic within 12 months before the entry of this study. Next, given that the maximum interval of the routine follow-up was 6 months, for ‘patients lost-to-follow-up’, ‘an adjusted follow-up duration’ was arbitrally defined as 12 months, twice as long as 6 months. Conversely, among patients who died during follow-up or whose last visit was within 12 months before entry to this study, the follow-up duration is an adjusted follow-up duration. In this study, 71 of the 290 (24.5%) patients were finally defined as patients lost to follow-up. When Cox analyses using a new concept of an adjusted follow-up duration based on all-cause mortality were performed again, only three variables, including male sex (HR 3.247, 95% CI 1.538, 6.852), FFS (HR 1.607, 95% CI 1.074, 2.406), and serum albumin (HR 0.399 95% CI 0.200, 0.793), were independently associated with all-cause mortality, which were similar to the results of Table 2 (Supplementary Table S1).

In terms of renal function impairment, this is a well-known critical risk factor for all-cause mortality in Korean patients [25]. Therefore, in this study, we defined renal function impairment as serum creatinine ≥ 1.7 mg/dL according to an item of renal insufficiency of the FFS [19] and performed additional Cox analyses with a categorical variable of serum creatinine ≥ 1.7 mg/dL, rather than a continuous variable of serum creatinine. Similarly to the main results, three variables, including male sex, the FFS, and serum albumin, were independently associated with all-cause mortality in patients with AAV but not serum creatinine ≥ 1.7 mg/dL (Supplementary Table S2). Additionally, in terms of internal validation, to increase the robustness of the results of this study, we performed bootstrapping for the internal validation of the model. Similarly to the main analysis, male sex (*p* = 0.002), the FFS (*p* = 0.041), and serum albumin (*p* = 0.012) were significantly associated with all-cause mortality as well.

Regarding the FFS, it was initially developed for the anticipation and prediction of poor prognosis and validated in patients with polyarteritis nodosa and EGPA [19,26]. However, there have not been a few studies reporting that the FFS assessed at diagnosis contributed to the prediction of poor outcomes during follow-up in patients with MPA and GPA to date [18,27]. Accordingly, we believed that the FFS would play an important role in anticipating the worse progression of AAV, and thus, in the present study, we included the FFS in variables at diagnosis, particularly, as one of the initial AAV-specific indices. On the other hand, if several already recognised poor or good prognosis factors for mortality such as old age, peripheral neuropathy, and interstitial lung disease, which have been suggested to date [28], had been included in the NFPM developed in this study, they might have made the NFPM more clinically useful. However, in the present study, if possible, we would like to maintain the current formula for the following reasons: (i) age was analysed together with other variables in the multivariable Cox analysis, but was excluded because it was statistically insignificant; (ii) major organ-specific risk factors are thought to be represented by the BVAS and FFS in the multivariable Cox analysis, because they are subitems of either the BVAS or FFS or both; and (iii) in this study, we aimed to present a method to derive a new formula composed of only significant variables through Cox analysis.

The greatest strength of this study is that it did not provide fixed indicators for all-cause mortality but rather suggested a method to develop a new formula using the initial independent predictors of all-cause mortality in an ethnicity/region-specific cohort of AAV. We are confident that the results of the present study may satisfy a need that could not be met by existing uniform mortality risks alone. Another strength of this study is that it includes a considerable number of patients. Given a previous study on Korean patients with AAV, it may be expected that approximately 2000 to 2500 patients are currently diagnosed with AAV. This study included 290 patients, which approximately ranges from 11.6% to 14.5% of the total expected patients. Therefore, we are confident that the results of this study could have significant representativeness in Korean patients with AAV.

This study has several limitations. First, this was designed and conducted as a retrospective study, and thus, it was not possible to strictly control the various confounding factors for all-cause mortality and thoroughly collect all clinical data before and at diagnosis. Second, even though we proposed the process to develop the adjustable formula of NFPM for ethnicity/region-specific patients with AAV, it should also be taken into account that it may be inconvenient to apply immediately due to an unfixed formula. Third, because NFPM values could not be measured and calculated serially, it was not possible to approach the more precise prediction of all-cause mortality by investigating the dynamic alterations of NFPM values. In addition, a close link was identified among several processes such as the aggravation of AAV, adverse events of immunosuppressive agents, of which the doses were escalated owing to MPA exacerbation, opportunistic infection related to immunosuppressive states, subsequent AAV aggravation due to recurrent infection, and progression to multi-organ failure. Therefore, as we could not clarify the causes of death, we used the term ‘all-cause mortality’ rather than ‘death’ in this study. We believe that a prospective future study with more patients will be more reliable and able to provide dynamic information on the predictive potential and clinical implications of developing a new formula using the initial independent predictors of all-cause mortality in an ethnicity/region-specific cohort of AAV.

## 5. Conclusions

In the present study, we demonstrated that it could be clinically useful and significant to develop a new formula to predict all-cause mortality using independent predictors in each different ethnicity/region-specific cohort of AAV. Therefore, we hope that the methodological approach of this study can be applied to patients with AAV from other races and regions.

## Figures and Tables

**Table 1:** Characteristics of 290 patients with AAV.

Variables	Values
**At diagnosis**	
**Demographic data**	
Age (years)	60.0 (49.8, 69.0)
Male sex (N, (%))	104 (35.9)
Body mass index (kg/m^2^)	22.6 (20.3, 24.6)
**AAV subtypes (N, (%))**	
MPA	160 (55.2)
GPA	74 (25.5)
EGPA	56 (19.3)
**ANCA positivity (N, (%))**	
MPO-ANCA (or P-ANCA) positive	198 (68.3)
PR3-ANCA (or C-ANCA) positive	48 (16.6)
Both ANCA positive	12 (4.1)
ANCA negative	56 (19.3)
**AAV-specific indices**	
BVAS	12.0 (7.0, 18.0)
FFS	1.0 (0, 2.0)
**Comorbidities (N, (%))**	
Type 2 diabetes mellitus	77 (26.6)
Hypertension	116 (40.0)
Dyslipidaemia	59 (20.3)
**Acute phase reactants**	
ESR (mm/h)	59.5 (22.0, 96.8)
CRP (mg/L)	13.8 (1.7, 64.9)
**Laboratory results**	
White blood cell count (/mm^3^)	9260.0 (6415.0, 12,942.5)
Haemoglobin (g/dL)	11.3 (9.5, 13.2)
Platelet count (×1000/mm^3^)	299.5 (228.0, 393.3)
Blood urea nitrogen (mg/dL)	17.8 (12.5, 31.0)
Serum creatinine (mg/dL)	0.9 (0.7, 1.8)
Serum total protein (g/dL)	6.8 (6.0, 7.3)
Serum albumin (g/dL)	3.7 (3.1, 4.2)
**During the follow-up duration**	
**All-cause mortality (N, (%))**	39 (13.4)
**Follow-up duration based on all-cause mortality (months)**	47.2 (14.5, 82.6)
**Medications (N, (%))**	
Glucocorticoids	282 (97.2)
Cyclophosphamide	161 (55.5)
Rituximab	49 (16.9)
Mycophenolate mofetil	55 (19.0)
Azathioprine	154 (53.1)
Tacrolimus	24 (8.3)
Methotrexate	24 (8.3)

Values are expressed as the median (25 and 75 percentiles) or number (N) (percentage). AAV: ANCA-associated vasculitis; ANCA: antineutrophil cytoplasmic antibody; ESR: erythrocyte sedimentation rate; CRP: C-reactive protein; MPA: microscopic polyangiitis; GPA: granulomatosis with polyangiitis; EGPA: eosinophilic granulomatosis with polyangiitis; MPO: myeloperoxidase; P: perinuclear; PR3: proteinase 3; C: cytoplasmic; BVAS: the Birmingham Vasculitis Activity Score; FFS: the five-factor score; ESR: erythrocyte sedimentation rate; CRP: C-reactive protein.

**Table 2:** Cox hazard model analyses of variables at diagnosis for all-cause mortality during follow-up in 290 patients with AAV.

Variables	Univariable			Multivariable		
	HR	95% CI	*p* Value	HR	95% CI	*p* Value
Age	1.062	1.031, 1.095	<0.001	1.024	0.991, 1.058	0.162
Male sex	2.663	1.410, 5.030	0.003	3.457	1.644, 7.270	0.001
Body mass index	1.121	1.026, 1.224	0.011	1.093	0.989, 1.208	0.080
MPO-ANCA (or P-ANCA) positive	1.438	0.713, 2.902	0.310			
PR3-ANCA (or C-ANCA) positive	0.674	0.263, 1.724	0.410			
BVAS	1.082	1.038, 1.129	<0.001	1.028	0.969, 1.091	0.354
FFS	1.960	1.446, 2.655	<0.001	1.655	1.094, 2.504	0.017
Type 2 diabetes mellitus	1.052	0.531, 2.081	0.885			
Hypertension	1.161	0.617, 2.183	0.644			
Dyslipidaemia	1.794	0.908, 3.545	0.093			
ESR	1.009	1.001, 1.017	0.036	0.995	0.983, 1.007	0.398
CRP	1.008	1.003, 1.012	0.001	1.000	0.992, 1.008	0.988
White blood cell count	1.000	1.000, 1.000	0.036	1.000	1.000, 1.000	0.681
Haemoglobin	0.790	0.682, 0.916	0.002	1.033	0.822, 1.300	0.779
Platelet count	1.000	0.998, 1.002	0.968			
Blood urea nitrogen	1.011	1.004, 1.019	0.004	1.004	0.989, 1.019	0.643
Serum creatinine	1.143	1.016, 1.284	0.026	1.014	0.826, 1.244	0.895
Total protein	0.562	0.386, 0.820	0.003	0.984	0.883, 1.096	0.765
Serum albumin	0.364	0.234, 0.567	<0.001	0.354	0.174, 0.720	0.004

AAV: ANCA-associated vasculitis; ANCA: antineutrophil cytoplasmic antibody; MPO: myeloperoxidase; P: perinuclear; PR3: proteinase 3; C: cytoplasmic; BVAS: the Birmingham Vasculitis Activity Score; FFS: the five-factor score; ESR: erythrocyte sedimentation rate; CRP: C-reactive protein.

## Data Availability

The data used to support the findings of this study are included within the article and the Supplementary Materials.

## Supplementary Materials

The following are available online at https://www.mdpi.com/article/10.3390/jcm14030779/s1, Figure S1: Comparison of the cumulative patients’ survival rates among NFPM value ≥ 2, male sex, FFS ≥ 2, and serum albumin ≤ 3.2 mg/dL; Table S1: Multivariable Cox hazards model analysis of variables with significance in univariable Cox analysis (adjusted follow-up duration); Table S2: Multivariable Cox hazard model analysis of variables with significance in univariable Cox analysis (serum creatinine ≥ 1.7 mg/dL).
