# Supplementary material for: A New Formula Consisting of the Initial Independent Predictors of All-Cause Mortality Derived from a Single-Centre Cohort of Antineutrophil Cytoplasmic Antibody-Associated Vasculitis
Source: J Clin Med. 2025 Jan 25;14(3):779. doi: 10.3390/jcm14030779 (PMC11818776; doi:10.3390/jcm14030779)
Supplement: Supplementary file 1 [file jcm-14-00779-s001.zip › SUPPLEMENTARY TABLE1(NFPM&AAV).pdf]

Supplementary Table S1. Multivariable Cox hazards model analysis of variables with significance in univariable Cox analysis (adjusted follow-up duration)

Variables	Multivariable		
	**HR**	**95% CI**	**P value**
Age	1.021	0.988, 1.056	0.213
Male sex	3.247	1.538, 6.852	0.002
Body mass index	1.089	0.984, 1.204	0.098
BVAS	1.023	0.966, 1.083	0.442
FFS	1.607	1.074, 2.406	0.021
ESR	0.996	0.984, 1.008	0.489
CRP	1.000	0.993, 1.008	0.944
White blood cell count	1.000	4.000, 1.000	0.793
Haemoglobin	1.005	0.805, 1.254	0.967
Blood urea nitrogen	1.002	0.988, 1.017	0.760
Serum creatinine	1.046	0.856, 1.279	0.660
Total protein	0.981	0.872, 1.105	0.756
Serum albumin	0.399	0.200, 0.793	0.009

AAV: ANCA-associated vasculitis; ANCA: antineutrophil cytoplasmic antibody; BVAS: the Birmingham Vasculitis Activity Score; FFS: the Five-Factor Score; ESR: erythrocyte sedimentation rate; CRP: C-reactive protein.
