# Supplementary material for: A New Formula Consisting of the Initial Independent Predictors of All-Cause Mortality Derived from a Single-Centre Cohort of Antineutrophil Cytoplasmic Antibody-Associated Vasculitis
Source: J Clin Med. 2025 Jan 25;14(3):779. doi: 10.3390/jcm14030779 (PMC11818776; doi:10.3390/jcm14030779)
Supplement: Supplementary file 1 [file jcm-14-00779-s001.zip › SUPPLEMENTARY TABLE2(NFPM&AAV).pdf]

Supplementary Table S2. Multivariable Cox hazards model analysis of variables with significance in univariable Cox analysis (serum creatinine ≥1.7 mg/dL)

Variables	Multivariable		
	**HR**	**95% CI**	**P value**
Age	1.023	0.990, 1.058	0.171
Male sex	3.443	1.650, 7.185	<0.001
Body mass index	1.080	0.973, 1.198	0.150
BVAS	1.030	0.971, 1.091	0.329
FFS	1.549	1.014, 2.365	0.043
ESR	0.996	0.984, 1.007	0.479
CRP	1.000	0.992, 1.007	0.913
White blood cell count	1.000	1.000, 1.000	0.651
Haemoglobin	1.058	0.842, 1.329	0.631
Blood urea nitrogen	0.999	0.985, 1.013	0.908
Serum creatinine ≥1.7 mg/dL	1.929	0.854, 4.493	0.112
Total protein	0.980	0.873, 1.099	0.725
Serum albumin	0.338	0.164, 0.695	0.003

AAV: ANCA-associated vasculitis; ANCA: antineutrophil cytoplasmic antibody; BVAS: the Birmingham Vasculitis Activity Score; FFS: the Five-Factor Score; ESR: erythrocyte sedimentation rate; CRP: C-reactive protein.
