# Supplementary figures and images for: A New Formula Consisting of the Initial Independent Predictors of All-Cause Mortality Derived from a Single-Centre Cohort of Antineutrophil Cytoplasmic Antibody-Associated Vasculitis
Source: J Clin Med. 2025 Jan 25;14(3):779. doi: 10.3390/jcm14030779 (PMC11818776; doi:10.3390/jcm14030779)

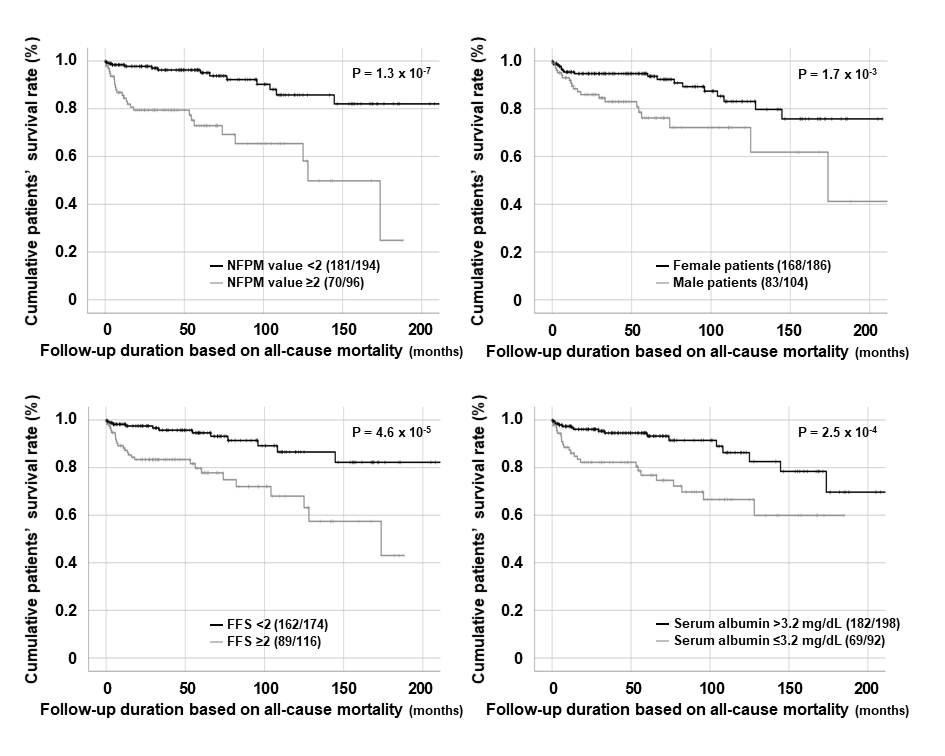

Supplement: Supplementary file 1 [file jcm-14-00779-s001.zip › SUPPLEMENTARY FIGURE1(NFPM&AAV).tif]
